# Tuberculosis and COVID-19 co-infection in Serbia: Pandemic challenge in a low-burden country

**DOI:** 10.3389/fmed.2022.971008

**Published:** 2022-11-16

**Authors:** Tatjana Adzic-Vukicevic, Maja Stosic, Gordana Antonijevic, Miroslav Jevtic, Aleksandra Radovanovic-Spurnic, Jelena Velickovic

**Affiliations:** ^1^Faculty of Medicine, University of Belgrade, Belgrade, Serbia; ^2^Clinic for Pulmonology, University Clinical Center of Serbia, Belgrade, Serbia; ^3^COVID Hospital “Batajnica”, University Clinical Center of Serbia, Belgrade, Serbia; ^4^Institute of Public Health of Serbia “Dr. Milan Jovanovic-Batut”, Belgrade, Serbia; ^5^Faculty for Health and Business Studies, Singidunum University, Valjevo, Serbia; ^6^Special Hospital for Pulmonary Diseases “Ozren”, Sokobanja, Serbia; ^7^Clinic for Infective and Tropical Diseases, University Clinical Center of Serbia, Belgrade, Serbia; ^8^Center for Anesthesiology, University Clinical Center of Serbia, Belgrade, Serbia

**Keywords:** tuberculosis, COVID-19, co-infection, Serbia, pandemic

## Abstract

**Introduction:**

COVID-19 and tuberculosis (TB) represent global threats to the public health system. The impact of COVID-19 on TB results in a reduction in the number of notified TB cases, delayed diagnosis and treatment, and increased case fatality and mortality rates. The aim of the study was to analyze the TB/COVID-19 co-infected cohort in Serbia as a low-burden country and compare it to the global TB/COVID-19 cohort.

**Methods:**

A retrospective analysis was done on 53 TB and COVID-19 co-infected patients treated in COVID hospital “Batajnica” in Belgrade and Special Hospital for Pulmonary Diseases “Ozren” Sokobanja in the period from 6 March 2020 to 1 April 2022. A comparative analysis with the global cohort published recently was also performed.

**Results:**

TB/COVID-19 cohort in Serbia included significantly fewer migrants and diabetes cases, but more cases with chronic respiratory diseases compared to the global. Descriptive analysis of TB cases in the Serbian TB/COVID-19 cohort showed fewer cases diagnosed with sputum smear and Gene Xpert/HAIN, fewer EPTB and mono-resistant cases, and more cases diagnosed with solid culture, unilateral pulmonary infiltrate (with bilateral cavity lesions), and bilateral pulmonary infiltrate (no cavities) compared to TB/COVID-19 cases worldwide. Nasal congestion and fever were more common COVID-19 symptoms in the global cohort. Radiology was more commonly used for the diagnosis of COVID-19 in Serbia. Typical bilateral ground opacities were less common among Serbian patients. Serbian patients spent fewer days in the hospital and achieved a higher PCR conversion rate and TB treatment success rate.

**Conclusion:**

The Serbian TB/COVID-19 cohort achieved a higher treatment success rate compared to the global cohort. Encouraging vaccination against SARS-CoV-2 for people with a current or past TB disease, as well as rapid diagnosis and targeted treatment of TB in highly specialized pulmonology institutions, presents key points to avoid excessive morbidity and mortality.

## Introduction

Tuberculosis (TB) presents a global public health threat with 10 million cases and 1.3 million deaths annually (1). Due to its rapid spread, clinical severity, and high mortality rate, with more than 6.4 million deaths in the past 2.5 years, the severe acute respiratory syndrome coronavirus 2 (SARS-CoV-2) pandemic represents the most contagious infectious disease worldwide (2). This epidemic had a great impact on all health systems in the world and the management of other diseases. The impact of COVID-19 on TB management is significant, especially in countries where the healthcare workforce has been reassigned to the COVID-19 emergency. This led to fewer TB diagnoses or diagnostic delays, and more TB deaths. However, not enough data on what actually happened are yet available. Diagnostic delays with more severe clinical presentations, worst outcomes, and missed follow-ups are only a few of the consequences and indirect effects of TB services disruption (3). A decrease in TB case notifications between 2019 and 2020 from 7.1 to 5.8 million cases was reported by the World Health Organization (WHO) (4). Results from the first pilot study of the Global Tuberculosis Network (GTN) on 49 TB/COVID-19 co-infected patients to the latest one of 767 patients from 34 countries showed that signs and symptoms are nearly the same (5, 6). Tuberculosis could be concomitantly diagnosed with COVID-19 and often presented with an increased case fatality rate. An estimated increase of ~20% in TB deaths is expected in the next 5 years (7). Due to the successful implementation of the WHO Directly Observed Treatment Short Course (DOTS) and STOP TB Strategy, the Republic of Serbia decreased its TB incidence rate from 37/100,000 to 9/100,000 in the past 17 years and now belongs to the low-burden TB country (8). The aim of the study was to analyze the TB/COVID-19 co-infected cohort in Serbia as a low-burden country and compare it to the published data on the global TB/COVID-19 cohort.

## Materials and methods

A retrospective analysis of 53 patients with active TB co-infected with COVID-19 and treated in COVID hospital “Batajnica” in Belgrade and Special Hospital for Pulmonary Diseases “Ozren” Sokobanja from 6 March 2020 to 1 March 2022 was performed. During the study period, we enrolled all patients of any age who were diagnosed and notified in these hospitals with active TB and COVID-19 simultaneously.

The data were obtained from medical records and include demographic data, laboratory, radiological, and clinical status at TB diagnosis and COVID-19 diagnosis, treatment, and follow-up. Follow-ups were done every month for each patient during the treatment period. After the treatment period, follow-ups were performed every 3 and 6 months, and some patients are still being followed up. On average, six to seven follow-ups were made per patient.

All data were compared to global TB and COVID-19 cohort publicly available data. Serbia has given its contribution to the global cohort with the first six patients (6).

Statistical analysis includes descriptive statistical analysis of all patients with details of TB and COVID-19 in our cohort. Variables were summarized using frequencies and percentages. Continuous variables with normal distribution were compared using *t*-tests, and categorical variables were compared using chi-squared or Fisher exact test.

## Result

The demographical, epidemiological, and clinical characteristics of the 53 patients with TB/COVID-19 are shown in Table 1. Most patients were men (66%, 35/54) with a median age of 45–64 years in 19 (38.8%) patients. Only 1 (2.0%) had a history of migration in the last 5 years. Human immunodeficiency virus (HIV) co-infected patients were not identified. Cardiovascular diseases, mostly arterial hypertension and chronic obstructive pulmonary disease (COPD), were the most commonly observed co-morbidities as summarized in Table 1. The TB/COVID-19 cohort in Serbia is composed of significantly fewer migrants and diabetes cases but more cases with chronic respiratory diseases compared to the global. According to vaccine status, only 8/53 (15.3%) of the study participants were fully vaccinated, including booster doses, with Sinopharm BBIP-CorV in 4 (7.5%), Pfizer-BioNTech in 3 (5.6%), and Astra Zeneca in 1 (1.9%) case.

**Table 1:** Characteristics of TB/COVID-19 cases in Serbia (*n* = 53) compared to the global cohort.

	**Serbian cohort**		**Global cohort**		**p**
**Characteristics**	***N***	**%**	***N***	**%**	
**Sex**					
Male	35/53	66.0	540/767	70.4	0.599
Female	18/53	34.0			
**Age category**					
18–44	18/53	34.0			
45–64	19/53	38.8			
65+	16/53	27.2			
**Immigrated in the last 5 years**	1/53	2.0	80/717	11.2	0.006
**Current smoking**	32/53	60.3	84/636	28.9	
**Concomitant diseases**					
Cardiac^*^	12/53	22.6			
Respiratory^**^	19/53	35.8	59/751	7.8	0.000
Diabetes^***^	9/53	17.0	157/753	20.8	0.404
Malignant^****^	2/53	3.7			
**Previous COVID-19**	0/53	0.0			
**Type of COVID-19 vaccine**					
Sinopharm BBIBP-CorV	4/53	7.5			
Pfizer-BioNTech	3/53	5.6			
Astra Zeneca	1/53	1.9			

* Arterial hypertension, ischemic heart disease, and cardiac insufficiency;

** chronic obstructive pulmonary disease, asthma, and respiratory failure;

*** all types of diabetes;

**** all localizations.

Among study participants, 9 patients (17.0%) had previously been treated for TB, while 19 (35.9%) had both diseases diagnosed at the same time and ~9 (16.9%) in the same week. As shown in Table 2, most of the patients had newly diagnosed TB (44/53, 83.0%). It was bacteriologically confirmed in 43/53 patients (81.1%), which was higher compared to the global cohort. Pulmonary localization of the disease was reported as well in more cases (52/53, 98%) in Serbia compared to global (612/732, 83.6). The majority of the patients (51/53, 96.2%) had pan-susceptible TB in Serbia. Mono-resistance to isoniazid was noticed in 2/53 (3.8%) of patients. Cavitary lesions were presented in statistically significantly more cases in Serbia compared to the global data, with 28/53 (52.8%) compared to 125/633 (19.7%), respectively.

**Table 2:** Descriptive analysis of TB in the Serbian TB/COVID-19 cohort compared to the global cohort.

		**Serbian cohort**		**Global cohort**		**p**
**Tuberculosis**		***N***	**%**	***N***	**%**	
History of previous TB treatment	New case	44/53	83.0	618/723	85.5	0.786
	Previously treated	9/53	17.0			
Anatomical site of the disease	PTB^*^	52/53	98.0	648/755	85.8	0.187
	EPTB^**^	1/53	2.0	189/738	25.6	0.000
TB laboratory confirmation	51/53	96.2	612/732	83.6	0.168
Radiology at TB diagnosis	Unilateral pulmonary infiltrate (with bilateral cavity lessons)	15/53	28.3	4/633	0.6	0.000
	Bilateral pulmonary infiltrate (no cavities)	24/53	45.3	108/633	11.7	0.000
	Unilateral pulmonary cavity lesion	13/53	24.5	121/633	19.1	0.217
	Pulmonary effusion	5/53	9.4			
Microbiology	Sputum smear	41/53	77.4	638/652	97.8	0.039
	Solid culture	47/53	88.7	441/638	69.3	0.019
	Liquid culture	20/53	37.7	24/638	50.9	0.064
	Gene Xpert/HAIN	10/53	18.8	410/638	64.5	0.000
Drug resistant pattern at TB diagnosis	Pan susceptible TB	51/53	96.2	517/607	85.2	0.233
	Mono resistant TB	2/53	3.8	90/607	14.8	0.004
	Multi drug resistant TB	0/53	0.0			
TB treatment regimens	HRZE	42/53	79.2			
	HRZES	8/53	15.1			
	RZES	1/53	1.9			
	RZE	1/53	1.9			
	HRE	1/53	1.9			
TB treatment outcomes	Cured and treatment completed	52/53	98.1	682/767	88.9	0.0345
	Died	1/53	1.9	85/767	11.1	

* Pulmonary TB;

** Extra-pulmonary TB.

The SARS-CoV-2 laboratory confirmation was available in all Serbian patients in a higher percentage compared to global as shown in Table 3. Most of our COVID-19 patients reported signs and symptoms of dry cough (37/53, 69.8%). Fever and shortness of breath were presented in one-third of the study participants (16/53, 30.2%) and (16/53, 30.2%), respectively. A statistically significant number of patients in our study underwent computerized tomography (CT) compared to the global cohort. Among the 18 (32.1%) CT-scanned patients, typical or atypical “ground glass” opacities were found (Table 4). In our study, bilateral ground glass opacities were found in a statistically significantly less number of patients (15.1%) compared to the global study (47.4%). Interestingly, 45% (24/53) of patients had TB diagnosed before COVID-19 (including nine patients with the previous TB) and 18.8% (10/53) of patients had COVID-19 diagnosed first.

**Table 3:** Descriptive analysis of COVID-19 in the Serbian TB/COVID-19 cohort compared to the global cohort.

		**Serbian cohort**		**Global cohort**		**p**
**COVID-19**		***N***	**%**	***N***	**%**	
**SARS CoV-2 laboratory confirmation**		53/53	100.0	23/763	94.8	0.593
**Signs and symptoms**	Fever	16/53	30.2	386/538	71.7	0.000
	Dry cough	37/53	69.8	311/538	57.8	0.114
	Shortness of breath	16/53	30.2	192/538	35.7	0.357
	Myalgia	5/53	9.4	87/538	16.2	0.091
	Nasal congestion	1/53	1.9	73/538	13.6	0.002
	Diarrhea	3/53	5.6	52/538	9.7	0.188
**Laboratory diagnosis**	PCR diagnosis	53/53	100.0	683/758	90.1	0.297
**Radiology at diagnosis**	CT scan	18/53	32.1	109/642	17.0	0.000
	Chest X ray	40/53	75.4	214/642	33.3	0.000
CT scan findings^*^	Typical ground opacity/opacities, unilateral (CT severity score 5–14)	10/53	18.8	40/266	15.0	0.327
	Typical Ground opacity/opacities, bilateral (CT severity score 15–25)	8/53	15.1	126/266	47.4	0.000
**Lung function tests at COVID-19 diagnosis**	Average sO2	95.4		96		0.951
**Hospitalization**	Average duration of hospitalization (days)	11.6		31.0		0.000
**PCR conversion rate**		53/53	100.0	271/474	57.2	0.000
**Treatment**						
Antivirals	Favipiravir	12/53	22.6	11/336	3.3	0.000
	Molnupiravir	17/53	32.1			
	Remdesevir	4/53	7.5	5/336	1.5	0.000
Immunomodulatory	Glucocorticoids	20/53	37.7	115/336	34.2	0.550
	Casirimibab/Imdevimab	1/53	1.9			
	Baricitinib	1/53	1.9			
	Sotrovimab	1/53	1.9			
Anticoagulants		35/53	69.8	94/336	27.9	0.000
Miscellaneous	Azithromycin	15/53	28.3	212/336	63.1	0.000

* Distribution of TB findings among patients who underwent CT examination is presented in Table 4.

**Table 4:** Distribution of TB findings among patients who underwent CT examination in the Serbian TB/COVID-19 cohort (*n* = 18).

**TB CT findings**	**COVID-19 CT findings**	
	**Unilateral ground glass opacities** **(*n* = 10)**	**Bilateral ground glass opacities** **(*n* = 8)**
Fibro caseous bilateral TB	1	1
Fibro caseous unilateral TB	2	3
Cavernous bilateral TB	2	1
Cavernous unilateral TB	1	1
Pulmonary effusion	4	2

All the patients with COVID-19 and TB in Serbia were hospitalized during anti-COVID treatment. The average duration of hospitalization among our patients was statistically significantly lower (11.6 days) compared to the global cohort (31.0). Pulse oximetry was performed in all patients, with an average oxygen saturation of 95.4%. Oxygen support *via* high-flow nasal cannula and later noninvasive ventilation was applied in one patient (1/53, 1.9%). Antiviral drugs including favipiravir, molnupiravir, and remdesivir were used in 33/53 (62.2%) patients. Favipiravir (22.6% compared to 3.3% globally) and remdesivir (7.5% compared to 1.5% globally) were used in our study in statistically significantly more cases than among the patients from the global cohort. Immunomodulatory treatment was applied in 23/53 (43.4%) patients; anticoagulation therapy in statistically significantly more cases in our study (35/53, 69.8%) than in 94/336 (27.9%) globally; and azithromycin in statistically significantly fewer cases (15/53, 28.3%) in our study compared to global (212/336, 63.1%).

The Serbian cohort achieved a statistically significantly better PCR conversion rate (100%) compared to the global cohort (57.2%), and TB treatment outcomes compared to the global cohort, namely, a treatment success rate of 98.1% compared to 88.9%.

## Discussion

Our study described and compared the findings of TB/COVID-19 co-infected patients in the Serbian cohort with a global cohort of 767 patients from 172 centers in 34 countries.

We found similarities in the results of our study with the results of the global cohort in terms of the male sex that can be attributable to the global predomination of the male sex among patients with TB (1). In contrast, we found low TB/COVID-19 co-infection among migrants compared to a higher number in the global cohort as well as data shown in other EU countries (9). Patients with COVID-19/TB had a much higher rate of comorbidities than patients with COVID-19 (10). Comorbidities may present additional risk factors for COVID-19 and tuberculosis. To date, evidence suggests that COVID-19 patients with preexisting comorbidities such as diabetes; hypertension; and cardiovascular, immunological, and respiratory diseases are at a greater risk for death (11). In our study, 42/53 (79.2%) patients had comorbidities. Compared to the global TB/COVID-19 cohort, COPD was more frequently found among respiratory concomitant diseases in our study because a large number of active smokers was found in our study group. Therefore, due to the high prevalence of both infectious diseases and due to worse prognosis of co-infection, further investigations on COVID-TB cases should be conducted (12). Some immunological findings suggest that TB disease may be transiently immunosuppressive. The combined effect of TB and SARS-CoV-2 infection likely causes a pronounced lymphocytopenia and, consequently, a CD4 + cell decrease as a reliable indicator of the severity of COVID-19. Different mechanisms of lymphopenia have been speculated, including lymphocyte death due to direct infection through receptor ACE 2, direct damage of SARS-CoV-2 to lymphatic organs, and lymphocytes deficiency induced by pro-inflammatory cytokines, such as tumor necrosis factor (TNF) α and interleukin (IL)-6 (13). In addition, COPD may be a risk factor for disease progression (14). Lung parenchyma damage due to pulmonary remodeling because of persistent cavitation, fibrosis, or bronchiectasis is present in approximately 50% of cured TB patients and might be associated with increased susceptibility to COVID-19 and a higher mortality rate (15). Both dual lung damages after TB and COVID-19 necessitate the follow-up of patients with post-tuberculosis lung disease who had COVID-19 pneumonia (16). Extrapulmonary tuberculosis (EPTB) was less frequently found in the Serbian TB/COVID-19 cohort compared to the global TB/COVID-19 cohort, suggesting a delayed diagnosis of TB because of lockdown and severely restricted access to tuberculosis diagnosis and treatment. In our case, we found hematogenous forms of tuberculosis (osteoarticular, genitourinary, and gastrointestinal), which have not been seen for a long time as reported in some other countries (3). Although not in high coverage, we found some patients vaccinated in contrast to the global cohort. The Republic of Serbia was among the first countries to start applying COVID-19 vaccines. At the moment of data collection, the following four COVID-19 vaccines were available in the country: BNT162b2 mRNA (Pfizer-BioNTech), Sinopharm BBIBP-CorV (Vero Cell^®^), Gam-COVID-Vac (Sputnik V), and ChAdOk1 nCoV-19 (AstraZeneca) (17).

We found differences in microbiological confirmation of the TB cases among our patients and the global cohort. Although Gene Xpert/HAIN is widely used in other countries, according to national and international guidelines, culture is used as the gold standard for TB diagnosis in low-TB burden countries (8). For those reasons, sputum smear was significantly more positive in the global vs. our cohort, solid culture was highly positive in ours compared to the global cohort, and Gene Xpert/HAIN was much more used in the global cohort. Drug susceptible pulmonary TB was found as the most common pattern of TB disease among our patients in line with the general population TB data (8).

The Serbian TB cohort reached a high treatment success rate due to prompt diagnosis and therapy, as well as a low frequency of mono-resistant and an absence of multidrug-resistant TB. A positive trend in TB declining in the past decade can be reversed by COVID-19, resulting in the reduction of tuberculosis testing and access to tuberculosis-related health services. Reports show that TB deaths would return to those seen in 2013 and estimated TB mortality could increase up to 20% between 2020 and 2025^1^ (7). Data from WHO and Stop TB Partnership predicted additional TB deaths between 2020 and 2025 (18). The impact of a pandemic on tuberculosis deaths during 2021 and 2022 is still unclear, probably worse because of fatal delta-driven surges and the current wave with an omicron variant. The COVID-19 pandemic affected tuberculosis preventive strategies, including a reduction of BCG vaccination by up to 60% in some parts of the world, treatment failure, and loss of follow-up (19).

The most common symptoms reported in both cohorts were dry cough, fever, and shortness of breath. Results correspond to global COVID-19 data^1^, while typical symptoms of COVID-19 like olfactory and taste disorders were not presented in our cohort. The mode of transmission of SARS-CoV2 and TB is the same, by small aerosol particles, but in COVID-19 also by large aerosol particles (droplets) (20). The initial signs and symptoms of COVID-19 and TB are like other respiratory infections, including influenza, and the main transmission route is through respiratory droplets, and the main targets are the lungs (5). Due to reported similar symptoms, for better screening, rapid molecular testing for TB and COVID-19 is suggested whenever it is possible (21).

Similar to symptoms, radiological findings overlapped in both COVID-19 and TB. The radiological findings of active tuberculosis and COVID-19 are often similar. Isolated upper-lobe pulmonary infiltrates are suggestive of TB, and lower infiltrates of other bacterial infections (22). Data showed that in almost half of patients co-infected with TB and COVID-19, CT findings were concluded only on COVID-19 (23, 24). COVID-19 radiological signs include typical or atypical ground-glass opacities. Existing evidence indicates that the features of lung imaging among COVID-19 patients include bilateral involvement, peripheral distribution, mixed ground glass opacities, and consolidations, while in COVID-19/TB pleural effusion, nodules and fibrosis could be present (25). We found the same distribution of unilateral while likely less frequent bilateral ground glass opacities since extensive bilateral CT findings including infiltrates and cavities accompanied by dyspnea are usually predictors of disease severity (9).

Due to the above-mentioned radiological overlapping in some of the patients, among some of the patients who had COVID-19 diagnosed before TB, we found cavities highly suspicious of TB, which were bacteriologically confirmed later. These data suggest that COVID-19 does not play a major role in advancing forms of TB (26). A potential connection between COVID-19 and active tuberculosis could be found in increased disease susceptibility and severity. Reduced frequency of *M. tuberculosis*-specific CD4+ T cells in peripheral blood was found in patients with COVID-19, suggesting increased susceptibility to progression to active tuberculosis (27). Diffuse lung parenchyma damage in patients with COVID-19/TB should be recognized in terms of invasive fungal infections, which are increasing problems in the long post-COVID period, especially in diabetic patients and patients on prolonged steroid use (28).

According to the COVID-19 treatment guidelines, treatment options include antiviral therapy, immunomodulatory therapy, and adjunctive therapy (29). Management of COVID-19 was similar in patients with both active tuberculosis and COVID-19 and COVID-19 alone. Antivirals including favipiravir, molnupiravir, and remdesivir were more frequently used in the Serbian TB/COVID-19 cohort compared to the global TB/COVID-19 cohort. Antiviral therapies may have a greater role in the early course of COVID-19 and influence good treatment outcomes of our patients, whereas immunomodulatory therapies are better in later stages (30). The need for steroids and oxygen support was confirmed due to oxygen saturation, partial pressure of oxygen, and clinical presentation. Glucocorticoids were used nearly the same in both cohorts. Multiple randomized trials suggest that systemic corticosteroid therapy improves clinical outcomes and reduces mortality in hospitalized patients with COVID-19 who require oxygen supplementation (31). According to the results of the same studies, larger samples are mandatory to explore the impact of corticosteroid therapy in patients with COVID-19/TB (32). If remdesivir is indicated for the treatment of COVID-19 in patients with active tuberculosis, clinicians should keep in mind that rifampicin could reduce the concentration of remdesivir (32).

The impact of COVID-19 and TB on long-term pulmonary sequelae and lung function impairment and the need for pulmonary rehabilitation should be determined in the next period (33).

The strength of our study is that it enrolled all diagnosed patients with TB/COVID-19 in our country. In addition, most of the variables are not collected in the routine COVID-19 and TB surveillance systems, and therefore the study gives a better insight into the characteristics of the patients with both diseases. The study limitation is that we were unable to perform adverse event analysis of therapies prescribed. Furthermore, it was not feasible to compare patients with TB/COVID-19 with the ones with a single disease alone.

## Conclusion

The Serbian TB/COVID-19 cohort achieved a higher treatment success rate than the global. A possible explanation could be the fact that all the patients with COVID-19/TB were first hospitalized in COVID hospitals and then transferred to a special hospital for TB because of monitoring and targeted therapy. Clinicians should be aware of both diseases because of overlapping signs and symptoms. The radiological presence of ground-glass opacities and rapid molecular testing is mandatory for the diagnosis of COVID-19. Comorbidities (particularly cardiovascular and respiratory) and male gender have an important influence on the morbidity of COVID-19/TB co-infected persons. Encouraging vaccination against SARS-CoV-2 for people with a current or past TB disease, as well as rapid diagnosis and targeted treatment of TB in highly specialized pulmonology institutions, presents key points to avoid excessive morbidity and mortality.

## Data availability statement

The raw data supporting the conclusions of this article will be made available by the authors, without undue reservation.

## Ethics statement

The studies involving human participants were reviewed and approved by Special Hospital for Pulmonary Diseases Ozren Sokobanja. The participants provided their written informed consent to participate in the study.

## Author contributions

TA-V and MS: concept and design and interpretation of the data. MS: statistical analysis. TA-V: drafting the manuscript. TA-V, MS, GA, MJ, AR-S, and JV: critical revision of the manuscript for important intellectual content. All authors have read and agreed to the published version of the manuscript.

## Conflict of interest

The authors declare that the research was conducted in the absence of any commercial or financial relationships that could be construed as a potential conflict of interest.

## Publisher's note

All claims expressed in this article are solely those of the authors and do not necessarily represent those of their affiliated organizations, or those of the publisher, the editors and the reviewers. Any product that may be evaluated in this article, or claim that may be made by its manufacturer, is not guaranteed or endorsed by the publisher.

## Footnotes

^1^https://www.cdc.gov/coronavirus/2019-ncov/symptoms-testing/symptoms.html.
